# Modelling of Environmental Ageing of Polymers and Polymer Composites—Durability Prediction Methods

**DOI:** 10.3390/polym14050907

**Published:** 2022-02-24

**Authors:** Olesja Starkova, Abedin I. Gagani, Christian W. Karl, Iuri B. C. M. Rocha, Juris Burlakovs, Andrey E. Krauklis

**Affiliations:** 1 Institute for Mechanics of Materials, University of Latvia, Jelgavas 3, LV-1004 Riga, Latvia; andrejs.krauklis@lu.lv; 2 Siemens Digital Industries Software, Via Werner von Siemens 1, 20128 Milan, Italy; abedin.gagani@siemens.com; 3 SINTEF Industry, Forskningsveien 1, 0373 Oslo, Norway; christian.karl@sintef.no; 4 Faculty of Civil Engineering and Geosciences, Delft University of Technology, P.O. Box 5048, 2600 GA Delft, The Netherlands; i.rocha@tudelft.nl; 5 Institute of Forestry and Rural Engineering, Estonian University of Life Sciences, 5 Kreutzwaldi St., 51014 Tartu, Estonia; juris.burlakovs@emu.ee

**Keywords:** polymer composites, fibre reinforced composites, biodegradable polymers, environmental ageing, durability, accelerated testing, modelling, lifetime prediction, superposition principles, creep, fatigue, plastic failure

## Abstract

Polymers and polymer composites are negatively impacted by environmental ageing, reducing their service lifetimes. The uncertainty of the material interaction with the environment compromises their superior strength and stiffness. Validation of new composite materials and structures often involves lengthy and expensive testing programs. Therefore, modelling is an affordable alternative that can partly replace extensive testing and thus reduce validation costs. Durability prediction models are often subject to conflicting requirements of versatility and minimum experimental efforts required for their validation. Based on physical observations of composite macroproperties, engineering and phenomenological models provide manageable representations of complex mechanistic models. This review offers a systematised overview of the state-of-the-art models and accelerated testing methodologies for predicting the long-term mechanical performance of polymers and polymer composites. Accelerated testing methods for predicting static, creep, and fatig ue lifetime of various polymers and polymer composites under environmental factors’ single or coupled influence are overviewed. Service lifetimes are predicted by means of degradation rate models, superposition principles, and parametrisation techniques. This review is a continuation of the authors’ work on modelling environmental ageing of polymer composites: the first part of the review covered multiscale and modular modelling methods of environmental degradation. The present work is focused on modelling engineering mechanical properties.

## 1. Introduction

Composite materials have been used more widely in engineering and product applications in the last decades, and this trend continues. The use of composite materials is expected to grow even more, mainly driven by two trends: (1) the requirements for reduced CO_2_ emissions, which can be partially achieved by reducing the mass of vehicles; and (2) new manufacturing methods, which can increase the production rate and reduce the unit cost for components, such as forming. Among the major benefits these materials offer are the high stiffness to weight ratio, which makes a strong case for the transportation industry, and the good durability, which has supported the use of composites in aggressive environments. Polymers and composite materials are often exposed to environmental influences such as water, humidity, elevated temperatures, pH, mechanical stress, and their combinations. Thus, environmental factors negatively impact the performance, affecting their durability [1,2,3,4]. Several reviews have been recently presented on the physical and chemical phenomena [2,5,6], including one from the authors [7].

Many engineering components are designed for a lifetime of several years or decades. Structural composites, such as fibre-reinforced polymers (FRP), are commonly employed in continuous applications spanning typically for 20–50 years and sometimes even longer, i.e., employed in the wind turbine, oil and gas, offshore and marine, aircraft, and transportation industries [1,8,9,10]. When designing such components, an important question is “how will this part perform in 10, 20, or 50 years?” To answer this question, we need to understand (i) which degradation mechanisms will affect the material and (ii) how can these degradation mechanisms be described mathematically and therefore predicted. Insufficient knowledge of the material behaviour under environmental impacts limits the full potential of composites and can result in oversizing components and, thus, an overall increase of the product cost. Hence, modelling the long-term durability of polymers and composites is highly interesting for both designers and end-users of novel materials and structures [3,11,12]. 

The design lifetime of structural composites is estimated based on short-term data using predictive models. Accelerated test methods (also called accelerated degradation tests) are testing programs that accelerate property degradation by subjecting it to conditions outside its normal service range [13,14]. In accelerated testing methodology, degradation is carried out in an intentional and controlled way aimed at effective reliability estimation in conjunction with modelling while reducing the time required for experimental testing [15,16]. The service lifetime is predicted by modelling the evolution of the critical mechanical characteristics (e.g., strength, stiffness) under accelerated degradation and establishing safety and reliability criteria. Polymer composites generally exhibit nonlinear and time-dependent behaviour that, combined with susceptibility to environmental degradation, makes it challenging to model their long-term performance [2,3,9]. This work provides a systematised approach to solving this challenge. Furthermore, the deployment of FRP in high-performance applications is relatively new compared to more traditional materials such as steel and aluminium. Thus, a complete understanding of FRP durability is yet to be achieved. 

Substantial gains and savings of resources of time and money can be gained through the use of modelling and simulation to understand material system performance [16]. Since for development of the new materials validation is expensive and time-consuming, the bottleneck is time and funding—modelling might be the way to replace testing programs, which would be beneficial for providing new innovative materials faster to the market [1]. A large percentage of a polymer’s development cost is determined by the decisions made early in the design process, which incorporates the testing phase. Implementing modelling and simulation reduces the number of prototypes needed because it can perform on-the-fly testing and product validation in a virtual space. Testing is the most time-consuming part of product development, and when failures occur, changes have to be implemented. Modelling and simulations would significantly reduce these costs [7,11,17]. 

**Figure 1:**
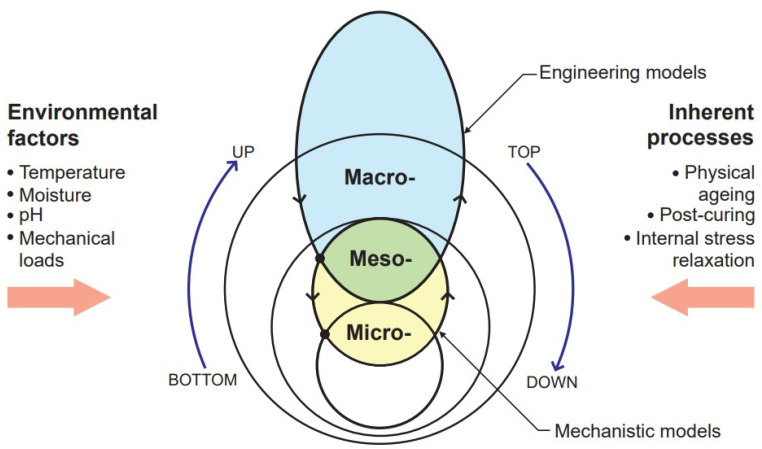
Schematic of models used for prediction of the durability of composite materials).

The degradation mechanisms depend on the interaction of the material system and the environmental conditions (Figure 1). Their action could be reversible (plasticization) or irreversible (damage, plastic strain evolution). In addition, polymer composites are subjected to inherent or “built-in” processes, e.g., physical ageing, post-curing, internal stress relaxation etc. Each deterioration mechanism involves several separate mechanisms that are accelerated with different rates and magnitudes. To account for such a complex phenomenon, modelling is performed on different scales of material structure: micro-, meso-, and macroscale (Figure 1) [18]. The models are correspondingly based on the degradation at (i) microlevel: composite constituents (polymer matrix and reinforcement) and their interaction (interphase), (ii) mesolevel: representative elements of a composite (e.g., ply in FRP laminate), and (iii) macrolevel: FRP laminate as a whole. Multiscale and modular modelling approaches employing data analysis from micro- to macrolevel of composites are therefore classified as “bottom-up” approaches. Despite the many advantages they provide [7], the main challenge is often related to a highly detailed knowledge of structure required as input data for modelling. Material characterisation at the microscale level is more complex than on the macroscale level.

Furthermore, idealisation of the actual structure of the material and discretisation of the degradation mechanisms can lead to the fact that the predicted degradation will differ from the experimentally observed global mechanism. Alternatively, material degradation can be assessed using a “top-down” approach. In this case, degradation on a microscale is explained from the analysis of macroproperties of a composite. From the point of view of engineering applications, the general behaviour of composite materials can often be characterised by less accurate (in terms of representative structure and specific properties) phenomenological and empirical models with a relatively small number of effective parameters. Such engineering models simplify reality and attempt to describe complex problems by simple rules. These are cost-effective solutions for the initial testing and design of novel composite materials with improved durability.

Durability prediction models are often imposed to conflicting requirements of versatility and minimum experimental efforts required for their validation. Substantial experimental and theoretical work has been performed to describe and predict the mechanical behaviour of polymer composites and failure under static and monotonic loads. The latest research in modelling and predicting creep and fatigue in polymer matrix composites is reviewed in [19,20]. Mathematical models not only predict durability but also contribute to the comprehension of the complex mechanical behaviour of composites. However, the inherent complexity of many composite materials results in complex mechanical models and a large number of experimental data required to determine material parameters. This essentially limits their use for practical applications. At the same time, engineering and phenomenological models, based on physical observations of composites macroproperties, are often manageable representations of complex mathematical models [18].

This work aims to provide a systematised overview of the state-of-the-art modelling tools for predicting the long-term mechanical performance of polymers and polymer composites under environmental impact. The mechanisms of environmental degradation on different structural levels and multiscale and modular modelling methods for prediction ageing effects in polymer composites were covered in the first part of the review, provided in [7]. This study covers durability prediction methods focusing on engineering mechanical properties, i.e., based on mesoscale to macroscale levels, distinguishing from mainly microlevel covered in the first part. In this context, ageing effects are manifest through changes in the mechanical properties of materials and predicted by the accelerated testing methodology. Limited to a global and homogeneous analysis, methods for predicting static, creep, and fatigue lifetime of various polymers and polymer composites under single or coupled influence of environmental factors are reviewed. Accelerated failure is predicted by means of degradation rate models, superposition principles, and parametrisation techniques.

The present review is aimed at scientists and industry professionals alike for purposes of accelerated testing, as well as for predicting the environmental durability of composites. It is a step towards fewer testing efforts to reduce substantially the costs of composite material qualification.

## 2. Models for Predicting Material Durability and Service Lifetime 

### 2.1. Rate Models 

#### 2.1.1. Arrhenius Model 

**Figure 2:**
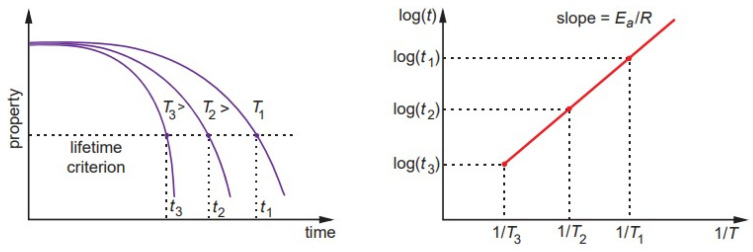
Lifetime prediction according to the Arrhenius model.

The Arrhenius model is widely used when temperature is the dominant accelerating factor in ageing. It is assumed that a single dominant degradation mechanism does not change during the exposure period, while the degradation rate is accelerated with an increase of exposure temperature [14]. The Arrhenius relation is given by

(1) $$K\left(T\right)=A\;\exp\left(-\frac{E_{a}}{RT}\right)\;or\;\;\ln K\left(T\right)=-\frac{E_{a}}{RT}+\ln A$$

where *K* is a reaction rate or degradation rate, *A* is a constant related to material and degradation process, *E_a_* is the process activation energy, *R* is the universal gas constant, and *T* is the absolute temperature. The degradation rate is proportional to the inverse time for degradation of a mechanical property for a given value set by the lifetime criterion, and log(*t*) vs. 1/*T* is a linear function with the slope *E_a_*/*R* (Figure 2). The Arrhenius relationship is widely used for lifetime predictions of polymers and composites through monitoring ultimate mechanical properties and their retention, e.g., tensile strength, interfacial shear strength, creep strength, and fatigue strength [6,21]. 

The durability prediction methodology in most studies is based on the time shift concept [8,21,22,23,24,25,26,27]. According to Equation (1), the time shift factor (*TSF*) for two different exposure temperatures $T_{1}$ and ($T_{1}\;$< $T_{2}$) can be calculated as

(2) $$TSF=\frac{t_{1}}{t_{2}}=\frac{A\;\exp\left(-E_{a}/RT_{2}\right)}{A\;\exp\left(-E_{a}/RT_{1}\right)}=\exp\left[\frac{E_{a}}{R}\left(\frac{1}{T_{1}}-\frac{1}{T_{2}}\right)\right]$$

Equation (2) has been further used in the predictive methods based on superposition principles and assessment of the temperature shift factors (Section 2.2). The activation energy in Equations (1) and (2) is commonly evaluated by thermal analysis methods, e.g., differential scanning calorimetry or thermogravimetric analysis by measuring heat capacity changes or mass losses under different heating rates. Alternatively, *E_a_* can be determined by dynamic thermal mechanical analysis (DMTA) assessing *T_g_* dependence on the test frequency [28,29]. 

Some recent studies considering the Arrhenius model and TSF approach for predicting the long-term strength of various FRP are listed in Table 1. Note that this methodology can be applied for assessment of tensile strength [22,30], interlaminar shear strength [23,26,27] or bond strength [25], under both static and fatigue loadings [26]. The accelerating temperature effect can also be coupled with other factors, e.g., absorbed water. For instance, Gagani et al. [26] applied the time shift concept to assess interlaminar shear fatigue lifetime of GFRP considering the effects of temperature and water immersion. The glass transition temperature of the material, and its decrease due to absorbed water, was used in Equation (2), enabling representation of both dry and water saturated samples in the same Arrhenius-based master curve. A detailed discussion on the fatigue of polymer composites is provided in Section 2.5.

#### 2.1.2. Eyring’s Model 

The reaction rate of a process can rely on several stressors. For example, nonthermal stresses such as humidity, voltage and mechanical stress may also play a significant role in accelerating degradation [14]. The Eyring model is based on chemical reaction-rate theory and describes how the rate of degradation of a material varies with stress. It is assumed that the contribution of each stressor to the reaction rate is independent; thus, one could multiply the respective stress contributions to the rate of reaction. The model is closely related to the Arrhenius model and is based on the fact that the logarithm of the reaction rate is inversely proportional to absolute temperature (Equation (1)). 

According to Eyring’s thermal activation flow theory, the strain rate (or the characteristic time) is given by the relationship

(3) $$\dot{\varepsilon }=\frac{1}{t}=A_{1}\exp\left(-\frac{E_{a}-\gamma \sigma }{RT}\right)$$

where *A*_1_ (s^−^^1^) is a material constant and γ is the coefficient linked to the activation volume; σ is the applied stress. Eyring’s activated flow theory is widely used to assess plasticity-controlled failure in thermoplastic polymers and composites [31,69,70,71,72,73,74,75,76,77,78,79,80]. A detailed discussion is given in Section 2.3.

#### 2.1.3. Zhurkov’s Model

Zhurkov developed the kinetic theory of strength of solids using temperature and tensile stress [32,81]. The relationship for calculation of the fracture lifetime $t_{f}$ is similar to Equation (3), while the coefficient γ referred to as the lethargy coefficient is linked to lattice structure and defects in it. In the general case of nonisothermal tests and σ varying in time (e.g., creep and fatigue tests), the fracture probability with account of the linear damage accumulation concept is given by the following equation [32,75,82]:

(4) $$\overset{t_{f}}{\underset{0}{\int }}\frac{dt}{t_{0}\exp\left(\frac{E_{a}-\gamma \left(\sigma \left(t\right)\right)}{RT\left(t\right)}\right)}=1$$

where $t_{0}$ is the time constant.

Zhurkov’s model initially developed for metals often could not give accurate lifetime predictions for polymers owing to the sensitiveness of their mechanical properties to strain rate and uncertain distinctions between the elastic and plastic ranges. As a result, the parameters involved in Equation (4) are interrelated stress, temperature, and strain-rate dependent functions. The kinetic concept of strength is applied to model fatigue damage evolution [32,83]. Hur et al. developed a modified Zhurkov’s fatigue life model introducing strain-rate-dependent lethargy coefficient and applied it to polypropylene reinforced with glass fibres [32]. The stress-based failure cycles in the ranges of low- and high-cycle fatigue were predicted and successfully validated by the proposed modified strain-rate model. The kinetic strength model applied to lifetime predictions of biodegradable polymers is discussed in the review paper by Laycock et al. [5]. For this type of material, Zhurkov’s equation is coupled with broader biodegradation models enabling assessment of stress effects on the lifetime via lowering the activation energy for chain scission.

### 2.2. Superposition Principles

Environmental ageing affects relaxation properties of polymers manifest in accelerated viscoelastic response (e.g., creep compliance, relaxation modulus) and time-dependent failure. This fact is widely applied to predict the long-term properties of polymer composites by using superposition principles and extending the time span. Depending on the accelerated factor, different methods are used: time–temperature superposition principles (TTSP), time–moisture superposition principles (TMSP), and time–stress superposition principles (TSSP). In other superposition principles, changes in the viscoelastic response are related to an ageing mechanism rather than a factor itself, e.g., time–physical ageing or time–curing degree [34,44,45,55,68], time–plasticization [28], time–hydrothermal ageing [46], and other superposition principles. Literature data on different superposition principles applied to various polymers and composites are summarised in Table 1.

Superposition principles are based on the assumption that time *t* and a factor *f*, which accelerates the relaxation processes (e.g., temperature *T*, moisture content *w*, and stress *σ*) are interrelated and interequivalent. The action of factor *f* leads to a parallel shift of the relaxation spectrum $\tau_{i}$ for a value $a_{f}$. For the reduced or effective time, this can be written as follows: $\tau_{i}^{\prime }=\tau_{i}/a_{f}$ or

(5) $$t^{\prime }=ta_{f}$$

where $a_{f}$ is the shift factor and f could be *T*, *w*, σ, or other. The concept of effective time allows one to correlate two time scales: the intrinsic time scale of the material revealed by viscoelastic relaxation and the observation time measured directly by a watch. Thus, it is possible to extend the observation time by influencing the intrinsic time by an external factor.

In a general case, the accelerating factor could change during the loading history, then the shift factor Equation (5) transforms into a time-dependent function as

(6) $$t^{\prime }=\overset{t}{\underset{0}{\int }}a_{f}\left[f\left(s\right)\right]ds$$

Under simultaneous action of several accelerated factors, e.g., $f_{1}\;\mathrm{and}\;f_{2}$, the total shift factor is contributed by both constituents:

(7) $$a_{f1,2}=F\left(a_{f1},a_{f2}\right)\;$$

Under additive contribution of environmental factors, i.e., when their action could be regarded separately, the time scale shift is realised by using a simple product of single shift factors, i.e., $a_{f1,2}=a_{f1}\times a_{f2}$. 

#### 2.2.1. Time–Temperature Superposition Principle

**Figure 3:**
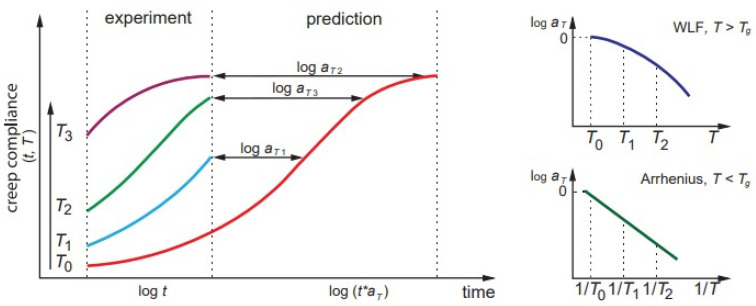
Superposition principles by the example of TTSP for creep compliance.

TTSP is one the most widely used prediction methods mainly due to its technical simplicity, controllability of testing procedures, and tractability of the obtained results. The schematic of long-term prediction of creep compliance by TTSP is shown in Figure 3. The viscoelastic property, e.g., creep compliances, at two different temperatures *T*_0_ and *T*_1_, differ only by a time scale defined by the temperature shift functions $a_{T0}$ and $a_{T1}$. Creep compliances *J* can reach the same values at different time moments *t*_0_ and *t*_1_, i.e., $J\left(t_{0},T_{0}\right)=J\left(t_{1},T_{1}\right)$ or according to Equation (5) $t_{0}a_{T0}$=$\;t_{1}a_{T1}$ and $\log t_{0}-\log t_{1}=\log a_{T1}-\;\log a_{T0}$. For simplicity, the reference shift factor is typically taken as unity $a_{T0}=1$. Thus, the creep compliance curves in logarithmic time axes are parallel for different *T* and shifted to each other for values $\log a_{T}$. The long-term prediction is represented by the generalised master curve (Figure 3). The concept of time-shift factors allows one to estimate the ratio between times required for a certain decrease in a mechanical property at two different operating temperatures [8,22]. Similar considerations are valid for other accelerated factors and related superposition principles. Applicability of TTSP has also been a validated methodology to determine the energy limit and stress threshold of linear viscoelastic behaviour of various polymers [37,38,84].

It should be noted that the procedure described is valid for thermorheologically simple materials exhibiting linear viscoelastic behaviour. However, it could be used in more complex cases after some modifications, e.g., vertical shift functions [57,85], stress-dependent parameters [35], equivalent strain rate [52], etc. The vertical shift factors could result from changes in the structure, e.g., degree of crystallinity in semicrystalline polymers [86] or residual curing in thermoset resins [39,87]. They can also be associated with the stress-dependent effects of nonlinear viscoelasticity [36]. Time–temperature shift factors are affected by the physical ageing of a material. As a result, superposition will not work if the testing time is comparable to the ageing time of the material. According to Barbero [88], experiments for TTSP should be performed on a timescale at least ten times shorter than the ageing time of the sample (see also Section 2.2.4). 

The mathematical relationships for the temperature shift functions depend on the temperature range considered. The Williams–Landel–Ferry (WLF) equation is valid for *T* between *T_g_* and *T_g_* +100 °C [89]:

(8) $$\log a_{T}=-\frac{C_{1}\left(T-T_{0}\right)}{C_{2}+T-T_{0}}\;$$

where $C_{1}$ and $C_{2}$ are material parameters. 

The Arrhenius equation is applied for $a_{T}$ calculations of glassy polymers at *T* < *T_g_* [89]:

(9) $$\log a_{T}=-\frac{E_{a}}{2.303R}\left(\frac{1}{T}-\frac{1}{T_{0}}\right)\;$$

where the temperature is taken in Kelvin and other symbols are the same as in Equation (1).

Equation (9) is sometimes used for $a_{T}$ calculations above *T_g_*, although with a different $E_{a}$ value, i.e., slope in $\log a_{T}\;\mathrm{vs}.\;1/T$ line (Figure 3). A generalised relationship for the time–temperature shift factor valid in a full operating temperature range is defined as follows [85,90]:

(10) $$\log a_{T}=\frac{E_{a1}}{2.303R}\left(\frac{1}{T}-\frac{1}{T_{0}}\right)H\left(T_{g}-T\right)\;+\left[\frac{E_{a1}}{2.303R}\left(\frac{1}{T_{g}}-\frac{1}{T_{0}}\right)+\frac{E_{a2}}{2.303R}\left(\frac{1}{T}-\frac{1}{T_{g}}\right)\right]\times \left(1-H\left(T_{g}-T\right)\right)\;$$

where *H* is the Heaviside step function; $E_{a1}$ and $E_{a2}$ represent the activation energies below and above *T*_g_, respectively.

As far as the shift factors are known, the lifetime of a polymer system *t* at an operating temperature *T* can be determined according to Equation (5) by the ratio [6]:

(11) $$t\left(T\right)=\frac{a_{T_{0}}}{a_{T}}\cdot t_{0}\left(T_{0}\right)\;$$

where $t_{0}\left(T_{0}\right)$ is the lifetime at the reference temperature. 

In the case of FRP, the temperature effect is associated with the viscoelastic properties of the polymer matrix [40,41]. Thus, the temperature shift factors usually are the same for the polymer and composite. This fact is employed in the accelerated testing methodology for the long-term durability of various FRP [42,43] (see also Section 2.5.2).

#### 2.2.2. Time–Moisture Superposition Principle

In TMSP, absorbed moisture (water) is considered a factor that accelerates the relaxation processes. The analogy between plasticizing effects of temperature and moisture on the viscoelastic behaviour of polymers is mentioned in several pioneering works: Onogi et al. [91], Maksimov et al. [92], Flaggs and Crossman [93], and Weitsman [94]. 

**Figure 4:**
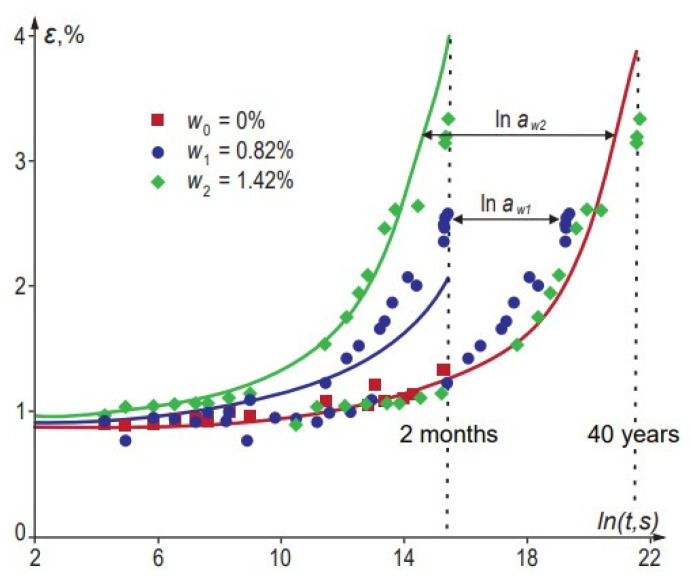
Creep curves of vinylester with different equilibrium moisture contents (*w*_0_, *w*_1_, *w*_2_) and the master curve constructed by applying TMSP. The Boltzmann–Volterra equation calculates the line for the linear viscoelastic solid and time–moisture shift function given by Equation (13). Data are taken from [50].

The general procedure for long-term prediction is similar to that in TTSP: short-term data are obtained at a fixed temperature for samples with different equilibrium water contents. The master curve is constructed by horizontally shifting these data to a reference curve (typically, a dry sample). An example of TMSP applied to long-term creep of vinylester resin is shown in Figure 4 [50].

The time–water content shift factor $a_{w}$ can be expressed by the relationship similar to the WLF equation (Equation (8)) [47,52]:

(12) $$\log a_{w}=-\frac{D_{1}\left(w-w_{0}\right)}{D_{2}+w-w_{0}}\;$$

where w and $w_{0}$ are water contents in the “wet” and reference states, respectively. $D_{1}$ and $D_{2}$ are material constants determined from experimental data; $\log a_{w}$ can also be given by a polynomial [44,50,51,95]:

(13) $$\log a_{w}=d_{1}\left(w-w_{0}\right)+d_{2}{\left(w-w_{0}\right)}^{2}\;$$

where $d_{1}$ and $d_{2}$ are empirical coefficients. 

The plasticizing effect of absorbed water is manifest as a *T_g_* drop. The Fox model [52,53], also known as Simha–Boyer model [96], is among the most known models used for the prediction of *T*_g_ variations with w:

(14) $$\frac{1}{T_{g}}=\frac{\left(1-w\right)}{T_{g0}}+\frac{w}{T_{g}^{H2O}}\;$$

where $T_{g0}$ and $T_{g}^{H2O}$ (−150 °C) are $T_{g}$ of the “dry” polymer and water, respectively. $T_{g}$ drop per 1% of absorbed water usually is in the range of 5–10 °C for epoxy systems [28,29,47,96] and 20–40 °C for polyamides [47,52]. 

Considering *T_g_* as an indicator of polymer chain mobility related to its free volume, Krauklis et al. developed the time–temperature-plasticization superposition principle [28]. The moisture (called plasticization) shift factors were determined by the Arrhenius-type equation (Equation (9)), changing operating temperatures to *T_g_* of the dry and plasticized ($T_{gw})$ polymer [28]:

(15) $$\log a_{w}=-\frac{E_{a}}{2.303R}\left(\frac{1}{T_{gw}}-\frac{1}{T_{g0}}\right)\;$$

An application on amine-cured epoxy validated the proposed method; this particular epoxy material system and the underlying mechanism of ageing are described in more detail in [97,98,99]. The activation energy $E_{a}$, determined by DMTA and assessment of *T_g_* changes with the test frequency, was found to be the same for dry and moisture-plasticized polymer. The moisture shift factors $\log a_{w}$ calculated by Equation (15) correlated well with those determined by a common shifting of the creep compliance curves [28].

By combining Equations (14) and (15), it is possible to calculate $\log a_{w}$ for material with any moisture content. The only parameters involved are $T_{g0}$ and $E_{a}$ that could be determined by thermomechanical methods without performing time-consuming creep tests. Then, according to Equations (11), (14), and (15), the lifetime of a plasticized polymer can directly be assessed from *T_g_* changes. 

TMSP has been applied for various polymers and polymer composites, and a list of some works is given in Table 1. The great majority of them are related to different epoxy systems [28,33,44,45,46,47,48,49] and epoxy-based composites [49,53], along with some other polymers, e.g., polyester [51], vinylester [50], and polyamide [47,52]. TMSP is often coupled with other superposition principles, e.g., TTSP, when studying temperature effects on dry and wet materials [28,52,53,66,67,100]. Some other rheological models that consider water plasticization effects on viscoelastic–viscoplastic behaviour epoxy and epoxy-based composites are discussed in [101,102]. Xiao and Li studied plasticizing effect of solvents on viscoelastic properties of gels and applied TTSP with the shift factor described by the WLF equation [103].

#### 2.2.3. Time–Stress Superposition Principle

**Figure 5:**
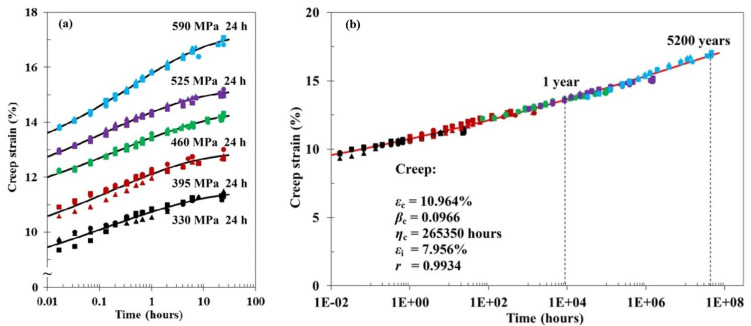
Creep curves for PA6,6 fibres at various creep stresses (**a**) and master curve obtained by TSSP (**b**). Adopted with permission from Ref. [59]. Copyright 2017 Willey.

The time–stress superposition principles (TSSP) allow accelerated testing of materials to determine their creep response and creep-rupture behaviour. The testing procedure is similar to that in TTSP (Figure 3), but the acceleration is obtained by increasing the stress instead of temperature. An example of TSSP applied to creep of PA6,6 fibres is shown in Figure 5: data of short-term tests are shifted to the master curve that gives the creep prediction for extremely long times [59]. TSSP is advantageous compared to TTSP because there is no need to use elevated temperatures, which may alter the chemical structure of the polymers tested. This fact is used in the related prediction technique, i.e., the stepped isostress method (SSM), allowing construction of the entire creep master curve by testing only one sample subjected to successively increasing stepped stresses [62,63,64,65]. At the same time, TSSP could have some limitations in terms of stress and time due to the differences in creep mechanisms under low and high loads.

Similarly to TTSP, the time–stress equivalence is based on free volume considerations assuming that the stress-induced change in the free volume fraction is linearly dependent on the stress change. A change in free volume affects the material mobility and, thus, its time-dependent mechanical properties [58,59].

The time–stress shift factor $a_{\sigma }$ is given by WLF-type (Equation (8)) relationship [104]:

(16) $$\log a_{\sigma }=-\frac{C_{1}\left(\sigma -\sigma_{0}\right)}{C_{3}+\sigma -\sigma_{0}}\;$$

where σ and $\sigma_{0}$ are actual applied and reference stresses, respectively. $C_{1}$ and $C_{3}$ are material constants determined from experimental data. The applicability of Equation (16) has been approved for various types of polymers and composites [36,58,59,61]. 

Alternatively, the relationship for $a_{\sigma }$ determination can be derived from the Eyring-type equation (Equation (3)). By comparing two different strain rates $\dot{\varepsilon }$ and $\dot{\varepsilon_{0}}$ for two different stress levels σ and $\sigma_{0}$, at the same temperature $T_{0}$, the shift function is given by the relation [42,62,63,64,65]:

(17) $$\log a_{\sigma }=-\log\frac{\dot{\varepsilon }}{\dot{\varepsilon_{0}}}=-\frac{B\left(\sigma -\sigma_{0}\right)}{2.303RT_{0}}\;$$

where *B* is a constant related to the activation volume.

Similarly to the WLF and Arrhenius equations for TTSP, the choice of the most applicable model for the stress shift function, Equation (16) or (17), depends on the material state and application. Based on their fundamental origin, Equation (16) is the most suitable when considering polymers in a rubbery state (*T* > *T_g_*), while Equation (17) is valid for glassy polymers (*T* < *T_g_*). TSSP could be applied to both creep and creep-recovery data. As demonstrated by the example of PA6,6 fibres [59], $\log a_{\sigma }$ is identical in both cases, pointing to linear viscoelastic behaviour. 

The coupled influence of stress and temperature on viscoelastic properties of various polymers and composites has been considered in several studies [36,56,58,61]. The combined time–temperature–stress superposition principles are elaborated based on an assumption on the additive contribution of both factors. One part of the short-term tests are conducted under constant stress and different temperatures, while the other is performed at a constant temperature and different stresses. Defining the temperature shift factor at a constant stress $a_{T}\left(\sigma_{0}\right)$ and the stress shift factor at a constant temperature $a_{\sigma }\left(T_{0}\right)$, the coupled temperature–stress shift function is given as follows [36,56,58,61]:

(18) $$a_{T,\;\sigma }\left(T_{0},\sigma_{0}\right)=a_{T}\left(\sigma_{0}\right)\cdot a_{\sigma }\left(T_{0}\right)\;$$

Based on Equation (18), the viscoelastic property functions, e.g., the creep compliances, in different thermomechanical states will have an equal value but different time scales. This can be written as follows [36,56,61]:

(19) $$J\left(T,\;\sigma ,t\right)=J\left(T,\sigma_{0},ta_{T}\right)=J\left(T_{0},\sigma ,ta_{\sigma }\right)=J\left(T_{0},\sigma_{0},ta_{T,\sigma }\right)\;$$

The benefit of the combined consideration of several factors is that it allows one to construct a master curve for a wide time scale and do it in one step via $a_{T,\;\sigma }$ instead of two steps via a combination of $a_{T}\left(\sigma_{0}\right)$ and $a_{\sigma }\left(T_{0}\right)$. However, one must be careful that the acceleration factor (temperature or stress) does not affect the physical or chemical characteristics of the material (e.g., by after-cure effects [34]) and its general deformation mechanisms (e.g., linear to nonlinear viscoelastic or viscoplastic behaviour). 

The temperature–stress shift function is derived based on free volume considerations and combining Equations (8) and (16) [58]:

(20) $$\log a_{T,\;\sigma }=-C_{1}\left[\frac{C_{3}\left(T-T_{0}\right)+C_{2}\left(\sigma -\sigma_{0}\right)}{C_{2}C_{3}+C_{3}\left(T-T_{0}\right)+C_{2}\left(\sigma -\sigma_{0}\right)}\right]\;$$

Equation (20) reduces to the WLF equation (Equation (8)) if there is no stress difference. 

Some recent studies considering TSSP alone [36,54,56,57,58,59,60] or coupled with other superposition principles [55,61] and applied to different materials are listed in Table 1.

#### 2.2.4. Time-Ageing Time Superposition

The long-term viscoelastic behaviour of polymers used under *T*_g_ is affected by physical ageing, i.e., a phenomenon related to the evolution of thermodynamic state manifesting as a reduction in free volume and changes in molecular configuration [105,106]. Structural rearrangements increase *T_g_* and material stiffening manifested via the increased strength and lower creep [44,96,107]. The time-ageing time superposition principles (TASP) are formulated considering the ageing time as a factor altering the relaxation spectra of a polymer (Equation (5)) [108]. Similarly to other superposition-based methods, time-dependence of material properties, which invalidates the use of Boltzmann superposition principles, is taken into account by applying the effective time-domain approach [108,109]. The real-time is normalised by the time-dependent relaxation time such that the relaxation dynamics remains invariant with respect to the effective time.

For studying the physical ageing phenomenon and applicability of TASP, samples are initially quenched from above *T_g_* to a temperature below *T_g_*. The time the material spends below its *T_g_* is referred to as the ageing time $t_{ag}$. Meanwhile, the temperature of ageing and the cooling rate are crucial parameters that determine the extent of physical ageing [107]. Short-term creep tests are conducted on samples with different $t_{ag}$ and the long-term master curve is constructed by horizontal shifting momentary data to a reference time $t_{ag0}$. The duration of these tests should be much shorter (at least by a factor of ten [88]) than the ageing time to exclude ageing effects during the test. The ageing shift factor $\log a_{ag}$ is expressed as follows [44,88,108,110]:

(21) $$\log a_{ag}=-\mu \log\left(\frac{t_{ag}}{t_{ag0}}\right)\;$$

where μ is the shift rate, 0<μ≤1 for most glassy polymers. The material does not exhibit physical ageing at μ=0. The μ value is related to the curing degree of a polymer and quenching parameters. Thus, the shift rate can be used as a viscoelastic screening parameter to select materials and their curing degree; μ is a function of temperature: it decreases down to 0 approaching material *T_g_*.

**Figure 6:**
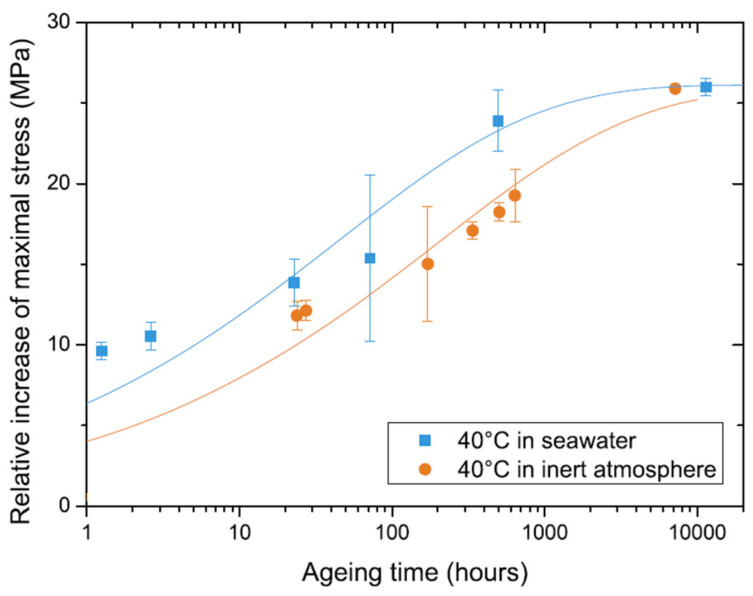
Strength vs. ageing time for amine-based epoxy conditioned in seawater up to saturation (wet) and in an inert atmosphere (dry). Adopted with permission from Ref. [96]. Copyright 2019 Elsevier.

Physical ageing is greatly affected by temperature and absorbed moisture due to material plasticization; thus, combined use of TASP and TTSP or TMSP is often considered [100,106]. Cross-coupled influence is accounted via the effective time approach, vertical shift functions, and temperature or moisture dependent shift rates [88,109]. Aniskevich et al. have studied the effects of ageing temperature and absorbed moisture on the physical ageing of various polymer matrixes in creep and stress relaxation tests [44,45]. Guen-Geffroy et al. investigated the coupling between physical ageing and water-induced plasticization in an amine-based epoxy [96]. The kinetic rate of physical ageing was found to be much faster in water due to the plasticization of the polymer. Figure 6 demonstrates differences in the strength evolution over ageing time for the dry and water-saturated polymer. 

Some authors correlate the material response with the time of any ageing process, e.g., caused by thermal or hydrothermal influence [46,55]. The time of a material exposure under specific conditions is considered as a factor affecting its relaxation behaviour. At the same time, the shift function in Equation (5) is related to this specific ageing time rather than temperature as in TTSP or moisture content in TMSP. For instance, the time–ageing time equivalence was applied to predict the viscoelastic behaviour of hydrothermally aged epoxy adhesive [46] and thermally aged PMMA [55]. Saseendran et al. [68] introduced the curing-time shift function to consider the curing history influence on the epoxy’s viscoelastic behaviour. The suggested approach combined with traditional TTSP is a powerful tool for predicting the long-term viscoelastic behaviour of partially cured polymer systems. However, the applicability of such ageing time-based approaches needs to be critically assessed due to the irreversible nature of ageing phenomena. Considering the cross-coupled influence of temperature and post-curing effects, a material needs to be classified as a thermorheologically complex material resulting in the development of more complex viscoelastic models and multistep procedures for generation of reliable master curves [55,68]. Some considerations on the curing-assisted chemical shrinkage and its effect on the viscoelastic behaviour of an epoxy system were reported by Böckenhoff et al. [35].

Temperature and time-related changes in the structure of polymers can lead to significant discrepancies between the time–temperature shift factors and master curves constructed based on the results of DMTA tests and traditional strain-controlled macrotests [34,111]. It is widely accepted that DMTA temperature and frequency scanning is an efficient approach, in terms of time and materials savings, for long-term predictions of the viscoelastic behaviour of polymer composites. In a recent study by Kontou and Spathis [111], the nonisothermal creep response of PMMA was predicted based on DMTA results. The authors introduced the energy barrier’s distribution density function determined from the experimental frequency-sweep data of the loss modulus. Once this function is determined, the fundamental time-dependent functions can be evaluated, and creep response can be effectively predicted. However, the approach has some limitations to the applied temperature and stresses to avoid the contributions of plastic strains. Another point resulting in discrepancies between the long-term predictions by different methods is related to a polymer’s physical ageing and after-cure effects during high-temperature accelerated DMTA tests or relatively long macrotests [34]. Duration of the former tests is generally in the range of tens of minutes, while the latter control tests can last for up to several months.

Guedes provided a comprehensive review of durability prediction methods of polymer matrix composites under static and fatigue loadings [9,75]. The lifetime predictions were critically assessed based on different failure criteria (rate theory of fracture, energy-based Reiner–Weissenberg criteria, fracture mechanics, Monkman–Grant).

### 2.3. Plasticity-Controlled Failure 

**Figure 7:**
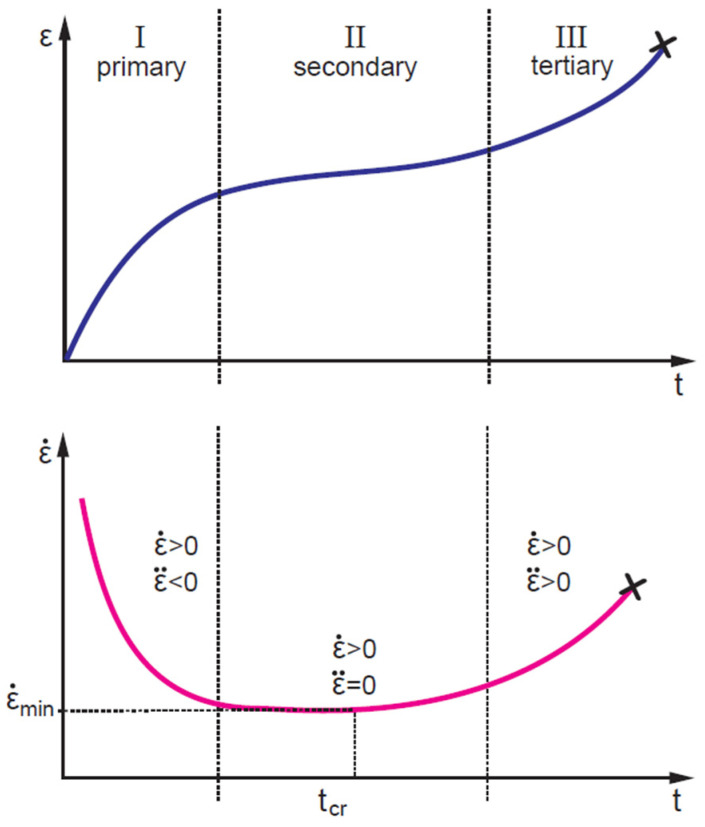
Typical creep curves: evolution of strain (**top**) and strain rate (**bottom**) with time.

Failure of polymers and other materials (e.g., metals, geomaterials, concrete) is associated with accumulation of (visco)plastic strains under loading. Creep failure testing is essential from the practical point of view for assessing the long-term static strength. Additionally, it contributes to understanding the mechanisms involved in the time-dependent deformation of the materials. The entire process of creep deformation can be divided into three stages: primary (transient), secondary (stationary), and tertiary (accelerated) creep (Figure 7). Primary creep is the viscoelastic region, where strain rate decreases with time and strain. During secondary creep, the strain rate reaches a constant steady plastic flow rate, which gradually accelerates (tertiary creep), eventually leading to strain localisation and failure [76,112].

Failure is an unstable process related to the microstructural specificity of a sample and testing conditions [31,80]; thus, failure predictions should be based on an extensive experimental data set and statistical analysis [113]. At the same time, in most applications, materials are exploited in the secondary creep regime, while tertiary creep is undesirable and considered the overshoot of the safety intervals [76]. Thus, many approaches consider the transition from the secondary to tertiary creep stage as a limiting factor or failure criteria defined by the strain rate minimum (Figure 7), for instance, the creep failure time model developed by Spathis and Kontou [31] and Monkman–Grant parametrisation [75,114] (see also Section 2.4). 

**Figure 8:**
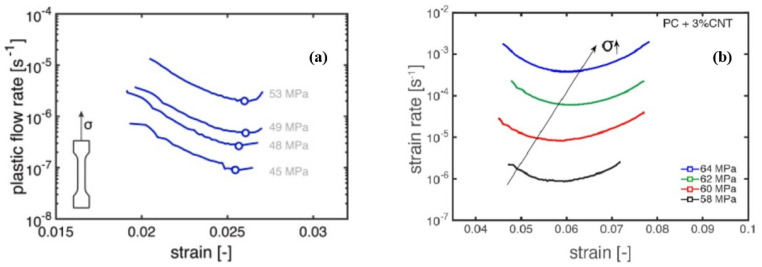
Sherby–Dorn plots for (**a**) glass-fibre reinforced iPP composites [72], and (**b**) polycarbonate/CNT composites [69], tested in uniaxial creep at 23 °C under various stresses.

Strain rate minima are determined from the strain rate vs. strain dependences called Sherby–Dorn plots [69,70,112]. As examples, Sherby–Dorn plots for glass-fibre reinforced isostatic polypropylene (iPP) [72] and carbon nanotube (CNT) reinforced polycarbonate [69] composites tested in uniaxial creep at 23 °C under various stresses are shown in Figure 8. It is seen that positions of strain rate minima increase with growing stress levels.

A methodology for prediction of long-term failure under the plasticity-controlled mechanism has been proposed by Erp et al. for oriented polypropylene [70] and further validated in a series of studies of Govaert and coworkers for various engineering thermoplastic polymers [73] and their fibre reinforced composites [71,72] and nanocomposites [69]. The method is based on the “critical strain” concept and the Eyring thermal activation theory for viscoplastic flow. This enables the assessment of the stress and temperature dependences of plastic flow under creep loading by means of tensile tests at different strain rates.

The Eyring’s relationship between the plastic flow rate ${\dot{\varepsilon }}_{pl}$ and the applied stress σ is given as follows [89]:

(22) $${\dot{\varepsilon }}_{pl}\left(\sigma ,T\right)={\dot{\varepsilon }}_{0}\exp\left(-\frac{E_{a}}{RT}\right)\sinh\left(\frac{\sigma \vartheta^{*}}{k_{B}T}\right)\;\;$$

where ${\dot{\varepsilon }}_{0}$ is a rate factor, $\vartheta^{*}$ is the activation volume, and $k_{B}$ is Boltzmann’s constant; other designations are the same as in Equation (3). Time-to-failure in the plasticity-controlled region can be estimated by calculating the total accumulated strain [69,70]:

(23) $$\varepsilon_{pl}\left(t\right)=\int_{0}^{t^{\prime }}{\dot{\varepsilon }}_{pl}\left(\sigma ,T,t^{\prime }\right)dt\;$$

where $\varepsilon_{pl}$ is the viscoplastic strain at a certain time, ${\dot{\varepsilon }}_{pl}$ is given by Equation (22). A criterion for failure is $\varepsilon_{pl}=\varepsilon_{cr}$, where $\varepsilon_{cr}$ is the critical strain related to the onset of plastic strain localisation.

According to the time–stress equivalence and experimental observations, the creep failure time multiplied by the strain rate at failure ${\dot{\varepsilon }}_{f}$ is constant for different applied stresses, i.e., ${\dot{\varepsilon }}_{f}t_{f}=const$ [115]. Taking into account considerations on the creep failure stages (Figure 7), the strain rate at failure ${\dot{\varepsilon }}_{f}$ could be replaced by the minimum strain rate ${\dot{\varepsilon }}_{min}$, i.e.,

(24) $${\dot{\varepsilon }}_{min}t_{f}=\varepsilon_{cr}\;$$

Going forward, Equation (24) is a special case of the Monkman–Grant relationship considered in the next section, Section 2.4.

It is worth noting that although $\varepsilon_{cr}$ is usually smaller than the actual failure strain (see strain values at ${\dot{\varepsilon }}_{min}$ in Sherby–Dorn plots, Figure 8), this phenomenological measure is reliable for predicting the time-to-failure of polymers [70].

Further, assuming that the state of deformation during secondary creep is identical to that obtained at the yield point $\sigma_{y}$ in a constant strain rate test, one can write the following [70]:

(25) $$\frac{t_{f}\left(\sigma_{1}\right)}{t_{f}\left(\sigma_{2}\right)}=\frac{{\dot{\varepsilon }}_{min}\left(\sigma_{2}\right)}{{\dot{\varepsilon }}_{min}\left(\sigma_{1}\right)}=\frac{{\dot{\varepsilon }}_{pl}\left(\sigma_{y2}\right)}{{\dot{\varepsilon }}_{pl}\left(\sigma_{y1}\right)}\;$$

**Figure 9:**
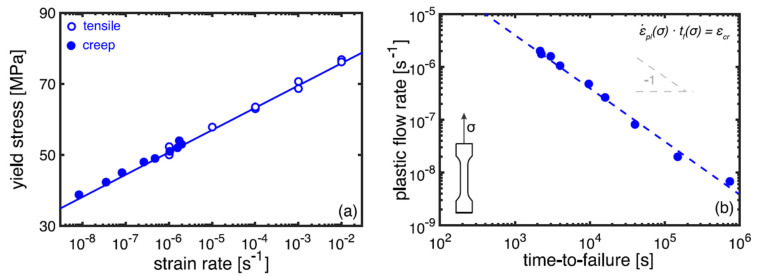
Strain rate dependencies of the yield stress in tensile tests and applied stress in creep tests (**a**) and a correlation between the plastic flow rate and time-to-failure according to Equation (24) (**b**) for glass fibre reinforced isostatic polypropylene composites [72].

The replacement of ${\dot{\varepsilon }}_{min}$ to ${\dot{\varepsilon }}_{pl}$ in Equation (25) is beneficial from a practical point of view, since the experimental assessment of ${\dot{\varepsilon }}_{pl}$ vs. $\sigma_{y}$ is based on the data of constant strain rate tests at different strain rates, which is much easier and less time-consuming compared to creep failure tests resulting in ${\dot{\varepsilon }}_{min}$ vs. σ dependences. A perfect correlation between the strain rate dependences of the yield stress determined in tensile tests and applied stresses in creep tests is demonstrated in Figure 9a for glass fibre reinforced thermoplastics [72]. According to Equation (24), the strain rate vs. time to failure follows a linear trend with a slope −1 (Figure 9b). 

The “critical strain” concept has also been verified for assessment of failure time of PA6 under the impact of temperature and humidity [74]. In addition, the method can be used as an effective tool for studying competition between two failure mechanisms in static loading and cyclic fatigue, namely plasticity-controlled and crack-growth controlled failure, respectively. Both mechanisms were effectively distinguished by comparing lifetimes for various thermoplastic polymer systems determined in creep and fatigue tests [71,72] (Table 1). “Critical strain” was independent of creep or fatigue loading under certain stress ratios. As applied to polymer nanocomposites, Pastukhov et al. demonstrated that adding carbon nanotubes into polycarbonate has a positive hampering effect on the plasticity-controlled failure. In contrast, the crack-growth-controlled regime has a negative accelerating effect [69].

**Figure 10:**
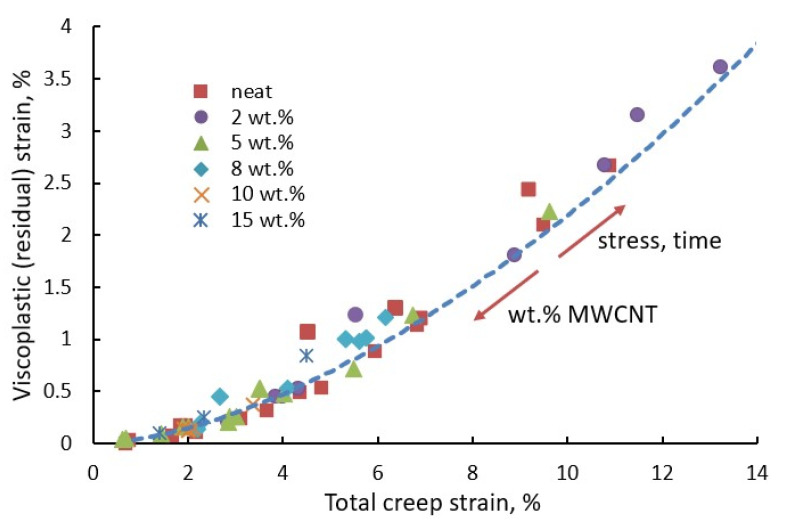
Residual recovery strain vs. total creep strain for polypropylene filled with different contents of MWCNT. Data obtained in creep-recovery tests under various loads and creep times; one point corresponds to one creep-recovery test. Data reproduced from [80].

Creep lifetime is strongly related to the accumulation of irreversible strains; thus, models for predicting their evolution are of great interest. The viscoplastic strain is expressed as nonlinear functions of stress, time, temperature, etc., e.g., Zapas–Crisman model discussed elsewhere [65,112,116,117]. Identification of multiple model parameters requires an extensive testing campaign that is often not justified in terms of costs. Simple, cost-effective methods with a minimum required amount of a priori known material parameters are advantageous for practical applications. In a recent study by Starkova et al. [80], a simple power-law relationship between the residual (viscoplastic) and the total creep strain was established (Figure 10):

(26) $$\varepsilon_{vp}=C_{0}{\left(\varepsilon_{creep}\right)}^{n}\;$$

where $\varepsilon_{vp}$ is the accumulated viscoplastic strain measured as the residual strain in creep-recovery tests, $C_{0}$ and *n* are material constants; *n* takes values in the range 0.7–2 and is equal to unity in the case of linear dependence between the residual and total creep strain [112,118]. The data were generalised for many polymers and composites reinforced with different types and amounts of fillers and tested under a wide range of stresses and temperatures. With increasing stress, loading time, temperature, or other external factors, one shifts forward on the curve $\varepsilon_{vp}$ vs. $\varepsilon_{creep}$, while increasing amounts of filler in host polymers (MWCNT in polypropylene in the present case) results in a shift down on the curve (Figure 10). Data representation in the form of Equation (26) is advantageous due to the implicit coupling of viscoelastic, viscoplastic, and damage-related strain components with no focus on the origin of irreversible effects. The results were consistent with known strain-rate-based modelling approaches [119,120].

### 2.4. Parametric Methods for Creep

Parametric approaches are methods through which the short-term creep-rupture data can be extrapolated using a time–temperature parameter. This concept is based on the assumption that all creep-rupture data can be superimposed to produce a single master curve: the stress vs. a parameter that combines time and temperature [114]. Long-term predictions are obtained based on this master curve constructed using available short-term measurements monitored in a few standard creep-rupture tests at different test temperatures and different stress levels. These extrapolation techniques were initially developed for metals and later validated for other materials, including polymers and composites [75,76]. It is worth noting that rupture can be defined by some limit value of strain, related to the safety criteria, or by actual rupture, depending on requirements set to a material.

**Figure 11:**
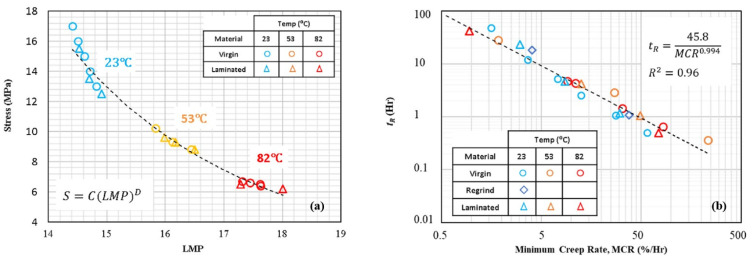
*LMP* master curve (**a**) and Monkman–Grant correlation $t_{r}$ vs. ${\dot{\varepsilon }}_{min}$ (**b**) for HDPE under various temperatures [57].

According to phenomenological models, Larson–Miller and Monkman–Grant parametrisations are among the most used formulations due to their simple form and tractability. In the former method, originally derived from the Arrhenius relation, the Larson–Miller parameter (*LMP*) relates the creep rupture time at different temperatures under given stress as follows [57,76]:

(27) $$LMP=T\left(\log t_{r}+C_{LMP}\right)\;$$

where *T* is the temperature in Kelvin, $t_{r}$ is the creep rupture time, and $C_{LMP}$ is a material constant. $C_{LMP}$ is determined by fitting a line $\log t_{r}$ vs. 1/*T* for a given stress level. Data for different temperatures in the axes stress vs. *LMP* fit on a common master curve (Figure 11) that could be linear [76] or fitted by a power law [57]. 

The Monkman–Grant (MG) parametric method uses the minimum strain rate ${\dot{\varepsilon }}_{min}$ as a key variable to assess the time to rupture $t_{r}$. It is assumed that the mechanisms that control creep deformation and creep rupture are the same to a large extent. The Monkman–Grant relationship is given by the following relationship [57,75]:

(28) $$C_{MG}=t_{r}{\left({\dot{\varepsilon }}_{min}\right)}^{\beta }\;$$

where $C_{MG}$ is the Monkman-Grant parameter and β is a material constant. Log–log plot of $t_{r}$ vs. ${\dot{\varepsilon }}_{min}$ forms a unified master curve for all temperatures (Figure 11), as it is demonstrated by the studies on HDPE [57], GFRP [75], and adhesive anchors [77] (Table 1). The practical advantage of the Monkman–Grant method, along with other strain-rate minimum based methods considered in Section 2.3, is that ${\dot{\varepsilon }}_{min}$ can be measured at an early stage of a creep test well before the material’s end-of-life, thus reducing the time required to predict the long-term time to rupture. 

The Monkman–Grant relation is a special case of the general Voight’s relationship describing rate-dependent material failure, which is a basis of the well-known failure forecast method originally developed for landslide and volcanic eruption forecasts [119,120]. Corcoran and Davies [120] analysed creep as a positive feedback mechanism showing that an increase in strain leads to an increasing strain rate, which indicates damage and proximity to failure. According to Guedes’s considerations on failure predictions of GFRP [75], the Monkman–Grant equation is “built-in” in the Reiner–Weissenberg energy criteria and maximum stress work criteria.

Both Larson–Miller and Monkman–Grant methods might offer the possibility of long-term extrapolation if the same creep-deformation mechanism operates during the whole creep life. If the dominant mechanism changes, measurements made at high stresses would not allow the prediction of the low-stress behaviour. Then, constants used in Equations (27) and (28) become stress and temperature-dependent functions, and thus, more materials need to be tested and examined using these techniques to generalise their use. The Larson–Miller approach is valid for assessing both static and creep rupture time, while the Monkman–Grant method cannot be used to compare creep failure with static failure under constant strain or stress rate [75]. The Larson–Miller parametrisation can also be used for predicting the fatigue lifetime of composites (see Section 2.5.3).

### 2.5. Fatigue Prediction Methods 

#### 2.5.1. Factors Affecting Fatigue Damage

Fatigue failure of composites is challenging to analyse due to the heterogeneous and anisotropic nature of the material, as well as the complexity and interaction of many damage mechanisms. Numerous mechanisms can be identified for traditional FRP laminates: matrix cracking, fibre-interface failure, fibre fracture, fibre microbuckling, crack coupling, and delamination [121]. The damage mechanisms interact with each other and are characterised by different growth rates; thus, these are associated with different stages of fatigue. Three general stages are typically observed in conventional quasi-isotropic laminates [122,123]: cycling loading initiates the formation of microcracks and voids (first stage), which are further localised, causing minor damage (second stage) and finally promote macrocrack growth leading to an ultimate material failure (third stage). This continuous damage process leads to significant degradation of the mechanical properties such as the strength and elastic modulus. Thus, these parameters are generally used as a measure of damage [121,124,125,126,127].

Fatigue durability is affected by numerous factors, which can be divided into three major groups:

(i) Material related factors: fibre type and dimensions, matrix type, fibre volume content, reinforcement structure (unidirectional, multidirectional, woven, braided, spatially reinforced, etc.), laminate stacking sequence, etc.
(ii) Testing related factors: loading conditions (stress ratio, cyclic frequency, monotonic/variable frequency, axial/multiaxial loading, force/displacement-controlled loading), and environmental conditions (temperature, humidity, water/salt water, UV).
(iii) Manufacturing and storage-related factors: manufacturing process, inherent defects and voids, thermal or ageing pre-history, etc.

Slight variations in the design of novel composite materials or their operating and storage conditions result in extensive growth of experimental testing campaigns required to characterise fatigue durability and assess their lifetime [128]. Fatigue models reduce the number of tests necessary for long-term predictions of the behaviour of composites under cyclic loading. The essential initial step is understanding the damage mechanisms occurring in composites during fatigue.

Many empirical and theoretical models based on both global (homogeneous) and micromechanical (multiscale) formulations have been established to model and eventually predict different material systems’ fatigue life. These are comprehensively discussed and systematised in numerous review papers [121,126,129,130,131,132,133,134]. The traditional models are often used in conjunction with statistical data analysis [135,136,137,138,139]. The present section is not aimed to give an in-depth discussion of the fatigue models but point out the main differences in existing modelling approaches that are crucial for fatigue prediction under environmental impact.

#### 2.5.2. Classification of Fatigue Models

Fatigue damage models may be assigned into different categories depending on their theoretical basis, “measurable” property, and structural levels of material involved in modelling [129]. There is usually no clear boundary between the categories, while categorisations are made mainly to highlight the role of the specific model in the development of subsequent models. Vassilopolous [130] reviewed the fatigue models for FRP in chronological order. Fatemi and Yang [131] categorised the reviewed theories and models into six categories: (a) linear damage rules, (b) nonlinear damage curve and two-stage linearization methods, (c) life curve modification methods, (d) approaches based on crack growth concepts, (e) continuum damage mechanics models, and (f) energy-based theories. Andersons [140] grouped the methods for fatigue prediction of composite laminates according to the structural level of material description: laminate, laminae, and fibre-matrix properties. Sendeckyj [141] introduced classification based on the fatigue criteria, namely four major categories: the macroscopic strength fatigue criteria, the residual strength and residual stiffness criteria, and the damage mechanism-based criteria. This classification with minor modifications has been further employed in numerous studies [121,142,143] and briefly justified within the following paragraphs.

**Figure 12:**
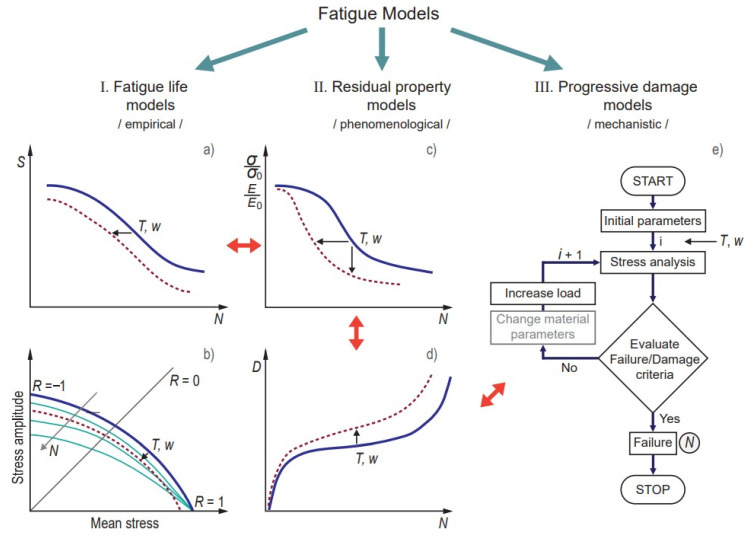
Classification of fatigue models and the principal ways for predicting the environmental impact (e.g., temperature *T* and water content *w*). Representative methods for fatigue analysis: (**a**) *S–N* curve; (**b**) constant life diagram; (**c**) residual strength/stiffness dependence on the number of cycles; (**d**) damage function; (**e**) flowchart of progressive damage analysis.

Based on Sendeckyj’s formulation, the fatigue models for polymer composites can be divided into three major categories: (I) fatigue life models, (II) residual property models, and (III) progressive damage models (Figure 12).

This classification consists of

(I) Fatigue life models (or empirical models) are based on the construction of the Wöhler *S–N* curves (Figure 12a), which provide information on the number of cycles (N) required to material failure under a given stress *S* and stress ratio defined as the ratio between the minimum and the maximum cyclic stress: *R*= σ_min_/σ_max_. Stress *S* may have different definitions: stress amplitude *S_a_* = (σ_max_ − σ_min_)/2, mean stress *S_m_* = (σ_max_ + σ_min_)/2, or normalised stress divided by a reference such as ultimate strength *S_u_*. Constant life diagrams (CLD), also called Goodman-type diagrams, are obtained by plotting *S_a_* vs. *S_m_* and presenting isolife lines with N=const, i.e., endurance limits (Figure 12b). Many examples of fatigue life models can be found in the literature [121,134]. Fatigue life predictions are commonly obtained by fitting a set of experimental data, in most cases by the Basquin-type power law equation [26,78,124]:

(29) $$S=A{\left(N\right)}^{-B}\;\to \;\log S=\log A-B\log N\;\;$$

where *A* and *B* are fitting parameters found from the *S–N* line in log–log (or linear–log) scale as the intercept at N = 1 (*A*) and its slope (*B*). Equation (29), however, is limited in use for high cyclic fatigue. In contrast, in low cyclic and very high cyclic fatigue tests, deviations from the linear *S–N* dependence typically take place due to fatigue–creep interactions or self-heating phenomena, respectively. Nevertheless, these models are considered the primary engineering models for predicting fatigue failure of composites due to their simplicity and easy tractability. The main drawback of the *S–N* models is that they do not indicate the underlying failure mechanism; thus, they are only valid for a specific material under specific loading and environmental conditions. A lack of data generalisation requires extensive testing campaigns for sufficient material characterisation. Note that in low-cycle fatigue applications or materials with significant plastic deformations, *S–N* curve stress-life analysis can be replaced by the strain-life analysis, whose mathematical representation is similar to Equation (29) [144,145]. 

(II) Residual property models (or phenomenological models) measure the loss of macroproperty during cycling loading. They can be subclassified in (i) strength and stiffness degradation models and (ii) fracture mechanics-based or crack growth models. In the former models, an empirical function is defined for describing experimentally observed gradual degradation of residual strength (σ/σ_0_) or stiffness (*E*/*E*_0_) with respect to the number of cycles (parameters with subscripts 0 are related to the initial undamaged materials property). The rate of strength degradation is typically defined as a function of several factors:

(30) $$\frac{d\sigma }{dN}=F\left(\sigma_{\min},{\;\sigma }_{\max},\;R\ldots \right)\;$$

Different forms of this function are considered in numerous research studies and reviews [121,125,126,136,140,146,147,148]. Failure occurs when the residual strength equals the maximum cyclic stress. Under constant-amplitude stress conditions, Equation (30) integration results in the *S–N* curve dependence. Thus, the fatigue life model is, in fact, a particular case of the residual strength model. The strength-based models provide a simple and clear explanation of fatigue failure. However, these are not widely accepted within the engineering community due to the extremely high experimental cost of measuring the residual strength (due to a large number of destructive tests, material- and testing-related sensitivity, etc.) [136]. Nevertheless, the strength degradation approach is advantageous in many cases due to the ability to account for the effect of fatigue damage without the need for its detailed analysis cycle-by-cycle.

The damage rate usually characterises the loss of stiffness:

(31) $$\frac{dD}{dN}=F\left(\sigma_{\min},{\;\sigma }_{\max},\;D\ldots \right)\;$$

where D is the damage variable defined according to continuum damage mechanics as $D=1-E/E_{0}$. The stiffness degradation models have been considered by numerous authors [121,127,149,150,151,152,153]. Contrary to the residual strength measurements, loss of stiffness presents much less data scatter and can be evaluated by nondestructive testing techniques, thus significantly reducing experimental costs for predicting fatigue durability of composites [140]. The residual stiffness and strength damage variables are also interrelated [154]. The limitation of the stiffness degradation model is the fact that it does not account for the different stages of composite damage mechanisms. For example, during the cyclic loading of off-axis laminates, initial stiffness degradation is caused by matrix transverse cracking while after that delamination and fibre failure occurs, which may have a different impact on stiffness degradation and residual life.

The fracture-mechanics-based models describe the initiation and growth of cracks in composites caused by cyclic loading [122]. Mostly, delamination cracks are under interest, although off-axis matrix cracking and fibre/matrix debonding can also be introduced into micromechanical models [139]. These models can also be classified as mechanistic models. The fatigue failure analysis is done by means of crack initiation curves, which is similar to an *S–N* curve (Equation (29)) but instead of the characteristic cyclic stress, the maximum applied energy release rate $G_{max}$ is used:

(32) $$G_{max}=A_{G}{\left(N\right)}^{-m}\;$$

where $A_{G}$ and *m* are material parameters. In the crack propagation phase, a power-law dependence, known as Paris law, is established between the crack growth rate da/dN and $G_{max}$ [71,123,139,155,156]:

(33) $$\frac{da}{dN}=C{\left(G_{max}\right)}^{p}\;$$

where *C* and *p* are material parameters. The fracture-mechanics-based models explain failure mechanisms and damage development in composites, although the general failure analysis is done by the empirical approach.

(III) Progressive damage models (or mechanistic models) estimate the current state of material degradation through a set of measurable internal damage variables (e.g., transverse matrix cracks, delamination). Generally, these models combine the phenomenological models and the definition of a fatigue failure criterion [155,157]. The progressive damage models calculate the stress analysis at each cycle or number of cycles and then recalculate the stress and internal variables according to the specific failure criteria [123,154,157] (see the flowchart in Figure 12e). The latter is defined depending on the nature and interaction of damage mechanisms. Thus, these models are expected to provide a deeper understanding of the fatigue failure phenomenon. However, this is done at the cost of complex numerical calculations.

Damage, e.g., fibre break and debond propagation, changes the local stress distribution, and this stress varies over the fatigue life [139]. To account for this, the concept of cumulative fatigue damage is adopted to sum the damage accumulation at different stress levels. Miner’s rule, also known as Palmgren–Miner rule or the linear damage rule, is the simplest and therefore most popular damage accumulation rule [129]. It defines damage of a structure subjected to cyclic loading as the linear sum of the ratios between the $n_{i}$ of cycles applied to the structure and the $N_{fi}$ of cycles that would cause fatigue failure of the structure on a given loading amplitude [158]:

(34) $$D=\underset{i}{\sum }\frac{n_{i}}{N_{fi}}\;\;\leq 1\;$$

where *D* is the damage variable, $n_{i}$ is the number of cycles at a given load amplitude and $N_{Fi}$ is the number of cycles that would cause the failure of a part under the same load amplitude. Damage must remain lower than 1 to avoid failure.

**Figure 13:**
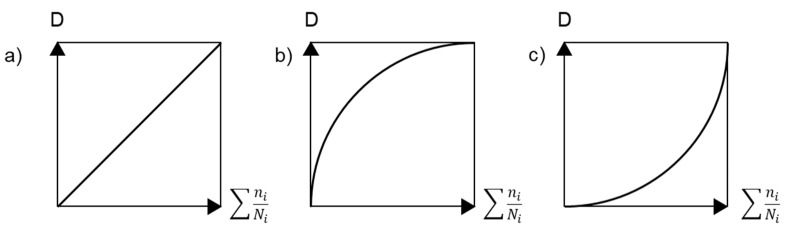
Damage accumulation types: (**a**) linear, (**b**) hyperlinear, (**c**) hypolinear.

This damage variable, as defined above, is clearly linear (Figure 13a). There are cases where it is useful to employ nonlinear damage laws to increase the damaging effect of low amplitude or high amplitude loading (Figure 13b,c). The damage parameter can be expressed through strength or stiffness changes that are correlated, e.g., by power-law dependence $D_{\sigma }={\left(D_{E}\right)}^{b}$, where *b* is the material parameter [154]. The progressive damage models are based on the strength or stiffness degradation concept, while property changes are assessed cycle-by-cycle. 

#### 2.5.3. Fatigue Prediction under the Environmental Impact

Variations in temperature, the humidity of ambient air, and other environmental factors contribute to the damage accumulation and determine the fatigue life of composites. Environments typically degrade the matrix material and the fibre/matrix interface, which are crucial for triggering further damage mechanisms and general fatigue response of FRP [26,132,156,159,160]. Reliable durability forecasts require a tremendous amount of highly undesirable testing costs. At the same time, studies on the fatigue of composites under environmental ageing are fragmentary and mostly experimental, while modelling approaches meet conflicting requirements of versatility and minimal experimental efforts needed for their validation. This implies the need to overview and systematise fatigue prediction methods under environmental impacts. Some of these methods are reviewed within the current section.

The predictive fatigue methodologies can be divided into several categories related to the classification used in Section 2.5.1 and the “property of interest” depending on how an environmental factor’s action is introduced into the model. Elevated temperature (*T*) and absorbed water (*w*) reduce fatigue lifetime of polymer composites that appears in a shift and/or change of slope of *S–N* curves (Figure 12a), narrowing and transformation of constant life diagrams (Figure 12b), decreased residual properties (Figure 12c), and accelerated damage (Figure 12d). The influence of accelerated factors can be accounted for by phenomenological approaches, e.g., TTSP, assessing global fatigue behaviour, and mechanistic models considering material damage on its different structural levels and updating the damage state until failure (Figure 12e). The current study is focused on phenomenological models due to their simplicity and availability for practical applications. Fatigue prediction methods can be grouped as follows:

(I) Construction of *S–N* master curves according to TTSP, similarly as it is done for viscoelastic properties of polymers (Section 2.2.1). The fatigue master curves are constructed based on Equation (29) and using the reduced frequency $f^{\prime }$ and the reduced time to failure $t_{f}^{\prime }$ concepts (Equation (5)):

(35) $$t_{f}^{\prime }=\frac{N}{f^{\prime }}=\frac{t_{f}}{a_{T}}\;$$

**Figure 14:**
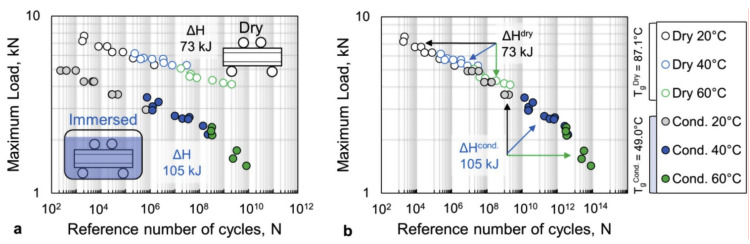
*S–N* master curves for dry and conditioned GFRP (**a**) and superimposed environmental master curve with the definition of equivalent temperature (**b**) [26].

For different temperatures, *S–N* curves are horizontally shifted to the reference *S–N* curve, typically obtained under room temperature. An example of *S–N* master curves constructed from four-point bending tests at different temperatures for dry and wet GFRP samples is shown in Figure 14 [26].

**Figure 15:**
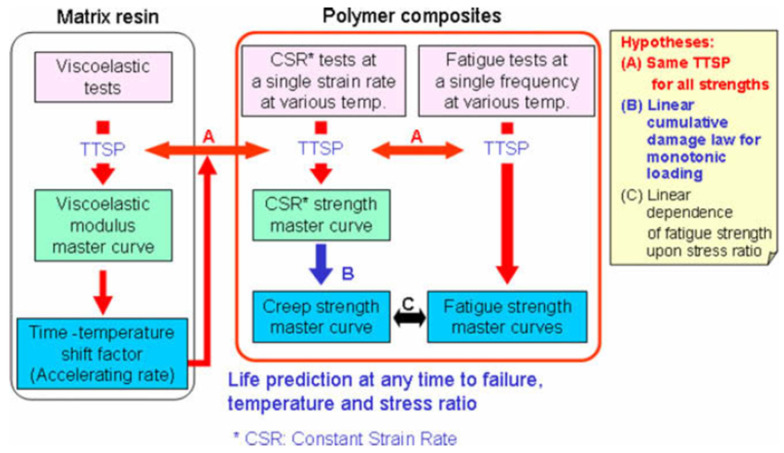
Formulation of accelerated testing methodology by Nakada and Miyano. Adapted with permission from Ref. [53]. Copyright 2009 Elsevier.

The shift factors are determined by the Arrhenius relationship (Equation (9)) with the activation energies different for temperature ranges above and below the polymer’s *T_g_*. Under the coupled influence of temperature and absorbed water, the water effect, related to both accelerated viscoelastic response of the polymer matrix and triggering additional damage mechanisms, is taken into account via the modified temperature shift functions. This methodology has been used by Gagani et al. [26], Zhou and Wu [133], and Fatemi et al. [146,161].

(II) Accelerated methodology developed by Miyano and Nakada et al. [42,53,90,162] assumes the same failure process for the accelerated loading history under elevated temperature and introduces three main hypotheses (Figure 15):

(A) The same TTSP is applicable for all strengths determined in constant strain-rate, creep, and fatigue tests. The temperature effect is solely associated with the viscoelastic properties of a polymer matrix; thus, the time–temperature shift factors are assumed to be the same for the polymer and composite and independent of a loading regime. 

(B) Linear cumulative damage law for monotonic loading predicts creep strength from the static strength master curve.

(C) Linear dependence of the fatigue strength upon stress ratio applies to predicting fatigue under an arbitrary stress ratio. 

The proposed methodology has been successfully validated for different FRPs, loading conditions, and ageing factors (temperature and moisture) [42,53,67,90,162]. Overall, the culminating point of this method also consists in the construction of *S–N* master curves, albeit at lower experimental cost than the previous method. Recently, authors proposed the advanced accelerated testing methodology for unidirectional CFRP introducing statistical assessment of the strength and a fatigue degradation parameter based on matrix viscoelasticity [43,135]. 

(III) Larson–Miller parametrisation for fatigue lifetime and construction of the master curves from data obtained at different temperatures, similar to the procedure for creep tests described in Section 2.4 (Table 1). According to methodology introduced by Eftekhari et al. [78,79], the fatigue stress amplitude can be expressed similarly to Equation (27):

(36) $$S=A\prime {\left(LMP_{f}\right)}^{B\prime }$$

where *A’* and *B’* are material parameters. The Larson–Miller parameter for fatigue ($LMP_{f}$) is defined as:

(37) $$LMP_{f}=\frac{T(\log t_{f}+C_{LMP_{f}})}{1000}$$

where $t_{f}$ is time to failure in fatigue test in hour, and $C_{LMP_{f}}$ is a material constant determined by fitting lines $\log t_{f}$ vs. 1/T for a given stress amplitude. The time to failure is converted to cycles to failure N using the test frequency: $t_{f}=N/\left(f\times 3600\right)$. 

**Figure 16:**
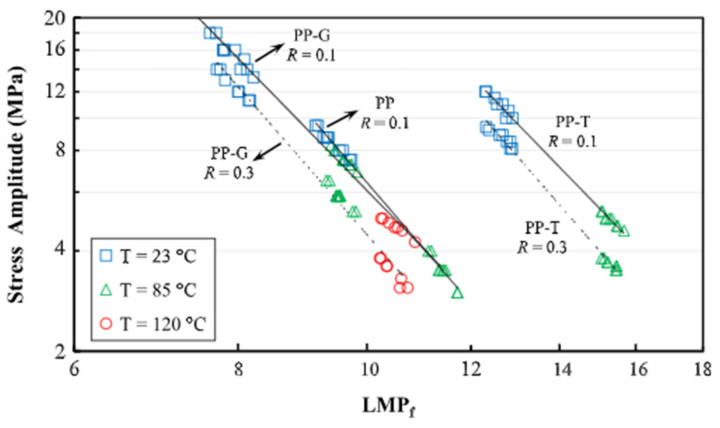
Larson–Miller master curves for polypropylene (PP), neat and reinforced with talc (PP-T), and glass fibres (PP-G) at *R* = 0.1 and 0.3. Adapted with permission from Ref. [78]. Copyright 2016 Elsevier.

Equations (36) and (37) are used to relate the stress amplitude, temperature, cycles to failure, and frequency for each material. The Larson–Miller fatigue master curves for polypropylene, neat (PP) and reinforced with talc (PP-T), and glass fibres (PP-G), are shown in Figure 16 [78]. Parameters $C_{LMP_{f}}$ were found to be independent of the stress ratio *R*, while *B’* varied slightly with *R* increase.

(IV) Normalisation of the characteristic stress of the fatigue models, e.g., stress amplitude in Equation (29) or residual strength in Equation (30), to the ultimate strength of an aged material rather than a pristine one: Such “normalised” *S–N* curves at different temperatures or ageing states of material are superimposed on each other. This observation comes from the fact that temperature (water) affects only the properties of a polymer matrix. At the same time, damage mechanisms under static and cyclic loadings of both the pristine and aged material are believed to be the same. The same hypotheses are used in the accelerated methodology by Miyano and Nakada [53].

Chamis and Sinclair [163] proposed a generalised empirical relationship for prediction of fatigue lifetime of hygrothemally aged graphite-fibre/epoxy-matrix composites:

(38) $$S=S_{u0}{\left(\frac{T_{gw}-T}{T_{g0}-T_{0}}\right)}^{1/2}-B\log N\;$$

where $S_{u0}$ is the reference initial static strength at the reference temperature $T_{0}$, *T* is the test temperature, and $T_{g0}$ and $T_{gw}$ are the glass transition temperature of the matrix in the dry and moisture saturated (wet) state, respectively; other parameters are the same as in Equation (29). Other authors modified Equation (38) to apply to thermoplastics by replacing glass transition temperatures to the melting [164] or other characteristic temperatures [165]. The first term in Equation (38) is associated with the constant *A* in Equation (29) and is related to the actual ultimate stress $S_{u}$ of the material at a given temperature and moisture content. Data plotted as $S/S_{u}$ vs. logN fit on a common dependence.

**Figure 17:**
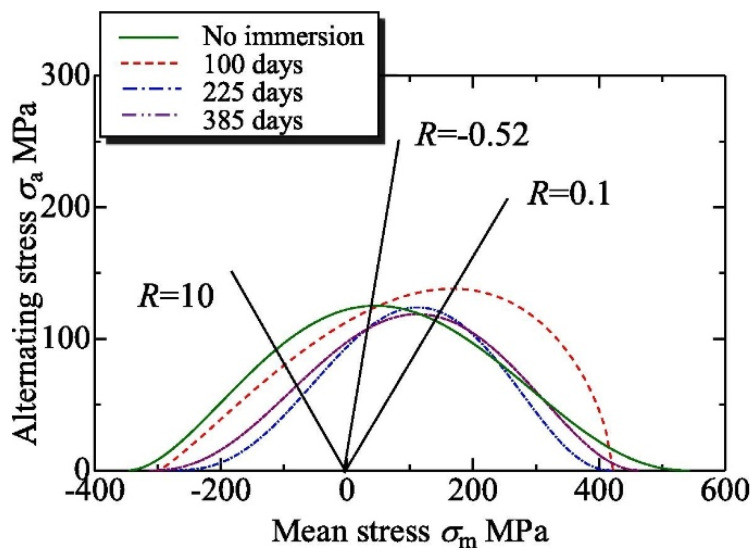
Constant life diagrams for plain-woven CFRP aged in seawater for different times. Adapted with permission from Ref. [167]. Copyright 2019 Elsevier.

The *S–N* “normalisation” is employed in numerous studies, e.g., [133,146,161,164,165,166]. It is simple, predicts conservative values, and should be adequate for preliminary designs. This method is often interrelated with the strength degradation concept described in the next paragraph. 

(V) Modelling *S–N* curves and the residual strength and stiffness by applying known models (Section 2.5.2) with temperature (or other environmental factors) dependent parameters: Such prediction methods can be based on empirical or physical considerations. The model parameters are determined by common fitting procedures, resulting in extensive and costly experimental testing. Figure 17 shows constant life diagrams for plain-woven CFRP aged in seawater for different times [167]. These were calculated based on the fatigue life prediction model using Basquin’s law and strength degradation model of Epaarachchi and Clausen. It is seen that environmental ageing resulted in lowering surface area under isolife lines indicating the decreased endurance limits of the material.

Although these approaches can contribute to understanding the damage mechanisms under the environmental impact, these are characterised by limited versatility since all the fitting parameters are specific for a given material only. Modelling of fatigue properties by this methodology has been used by Tang et al. [151], Khan [152], Cormier et al. [136], Mivehchi et al. [149], Koshima et al. [167], Eftekhari and Fatemi [78], Amjadi and Fatemi [166], Prabhakar et al. [145], Solfiti et al. [168], and Acosta et al. [169]. The results are summarised in Table 2 regarding the environmental factors, material and testing parameters, and general concepts employed in modelling. Some alternative approaches for predicting fatigue lifetime under the influence of environmental factors are presented in [102,137,156,160].

## 3. Discussion 

Many approaches have been proposed for predicting the long-term properties of polymer composites based on short-term tests allowing for reduction of the time scales and costs required to obtain long-term data. Accelerated testing methodologies enable us to reproduce natural exposure effects in short observation times. The problem is that the acceleration factors are often excessively amplified so that the degradation scenario may not correspond to the actual situation, and models give misleading forecasts [2]. In the best case scenario, this leads to overestimated safety intervals and oversizing of composite components, while in some cases it could even lead to an underestimation of safety intervals. Most studies consider environmental ageing within the timeframe of days and months, rarely years, and very rarely decades [170,171]. Another factor affecting the reliability of model predictions is related to neglecting synergetic effects from simultaneous action of several accelerated factors. There is still a great uncertainty on the long-term evolution of material properties due to limited research on comparing results obtained in laboratory conditions and after actual exposure. Collecting and systematising experimental results on the durability of different composites under the uncoupled and coupled influence of environmental factors is highly advisable for establishing empirical correlations and increasing the reliability of long-term forecasts.

The development of several degradation processes accompanies environmental ageing. The deterioration mechanisms can be interrelated and accelerated with different rates and magnitudes. To describe such a complex phenomenon, modelling is performed on different scales of material structure: micro-, meso-, and macroscale (Figure 1). Mechanistic models provide accurate predictions, although the main problem remains having a very detailed knowledge of the material structure. Engineering models simplify reality and describe complex problems by simple rules. These are based on general observations of the material performance at the macroscale and thus involve a relatively small number of effective parameters. The use of complex mechanical models with more accurate formulations (in terms of representative structure and specific degradation mechanisms) is not always justified from the point of view of practical applications. Engineering models are used as cost-effective solutions for the primary design of novel composite materials. The use of excessively sophisticated and thus complex mechanistic models requiring large amounts of experimental data sets for implementation, which does not necessarily pay off in terms of accurate predictions. In addition, there is no need to put design specifications to a higher accuracy level than required, especially if less accurate models still provide sufficiently reliable and valuable results. 

Vassilopolous [130], in his critical review on fatigue modelling, stated that many models for fatigue life are almost equivalently reliable. Despite mechanistic or phenomenological origin, most models are more likely to be classified as empirical since numerous model parameters related to microstructure are found by fitting the experimental curve of a “macroproperty”. The author notes that most of the investigations that proposed models simply fit them to the available experimental results, with their predictive ability remaining uncertain. Similar notes can be applied to many complex viscoelastic–viscoplastic models used for creep prediction. Another critical moment is associated with the probabilistic nature of failure. Therefore, ultimate properties and lifetimes of composites should be analysed statistically due to the significant variations exhibited during testing.

It is interesting to link the present discussion with that of the first part of this review effort [7]. The discussion in [7] focuses primarily on lower-scale material models to describe diffusion, reaction, and the resulting degradation of constituent materials (i.e., fibres, resin, and interphases) and on modular/multiscale numerical frameworks for upscaling these phenomena to higher scales of interest. In contrast, the focus here lies on phenomenological efforts to directly describe the mechanical degradation of composite materials at the mesoscale. Apart from being related alternatives to model ageing at the mesoscale, the modular paradigm and multiscale techniques in [7] can be further combined with the models discussed here in order to upscale degraded mechanical properties from the mesoscale up to even higher scales of interest (e.g., component scale, structure scale). Furthermore, the current trend in computational mechanics to employ machine learning for surrogate modelling [172], model selection [173], and data assimilation [174], which was briefly described in [7], is expected to also profoundly impact the class of models treated in the present discussion.

The present review considered general durability prediction methods with no focus on any specific material. Although model validations have mainly been performed on traditional polymers and polymer composites, many of the models considered can be applied to other classes of materials, e.g., wood, concrete, alloys, etc. Uncertainties may arise with durability modelling of novel materials that recently appeared in the composite market, e.g., biodegradable polymer composites, nanocomposites, 3D printed materials, shape-memory, functionally graded, and metamaterials [175,176,177,178]. Owing to the complex heterogeneous structure or “built-in” degradation or gradient properties of such materials, their mechanical performance cannot be predicted by traditional methods. Further research is needed on this subject. Regarding biodegradable polymer materials, durability and lifetime prediction techniques have been collected in several reviews [5,6,179]. However, they are mainly focused on predicting the end of life of materials and not service lifetime and mechanical properties. 

Environmental degradation has a “two-edged sword” nature: degradation is unfavourable for predicting service lifetimes and favourable for predicting end-of-life of materials. A deep knowledge of the degradation mechanisms can enable the design of products that degrade at the end of their intended lifetime, reducing their environmental impact. These points were discussed in the first part of the review [7]. Material designers face the question of what is more important, how the environment impacts material properties or how the material impacts the environment. The choice depends on the application of the material. In the case of short-term use products, preference will be given to biodegradable polymer composites due to their short end-of-life and climate neutrality [180,181]. In long-term structural applications, when the key factor is high durability and long service lifetime, preference will be given to traditional high-performance composites paying less attention to the environment-friendliness of these materials. The latter fact leads to a necessity to explore and find appropriate routes to handle composite waste such as recycling [182]. Overall, management of plastic wastes, reuse, and recycling of plastics is in accord with the EU plan for 2030 to avoid leakage of plastic into the environment and promote reuse and recycling [183]. 

## 4. Conclusions

This review provides a systematised overview of the state-of-the-art modelling tools for predicting the long-term mechanical performance of polymers and polymer composites under environmental impact. This is a continuation of the authors’ work on modelling environmental ageing of polymer composites: the first part of the review covered multiscale and modular modelling methods of environmental degradation as the physical and chemical phenomena considering materials on micro- and mesoscale. The present review focused on prediction of durability of polymer composites by engineering models, i.e., modelling of their macroproperties. Accelerated testing methods were analysed for predicting static, creep, and fatigue lifetime of various polymers and polymer composites under the single or coupled influence of environmental factors. Service lifetimes were predicted by means of degradation rate models (Arrhenius, Eyring, and Zhurkov), superposition principles (time–temperature, time–moisture, time–stress, time–ageing, and superposition principles) and parametrisation techniques (Larson–Miller and Monkmann–Grant). The role of plasticity-controlled failure in creep and fatigue tests was discussed. Fatigue models were systematised according to their theoretical basis and a method used for accounting for environmental effects. Some current challenges associated with durability prediction of advanced polymeric materials were briefly highlighted. The review article is useful for scientists and the industry alike for purposes of accelerated testing, and for predicting the environmental durability of composites. The models and methods covered in this work provide cost-effective engineering solutions for the initial testing and design of novel composite materials.

## Figures and Tables

**Table 1:** A condensed list of recent works on methods for predicting long-term mechanical properties of polymers and polymer composites.

Prediction Method	Material	Property	Ref.
**Rate models**			
Arrhenius model	GFRP	Tensile strength	[22,30]
	GFRP	ILSS	[27]
	GFRP	Fatigue ILSS	[26]
	GFRP bars	Tensile strength	[8]
	CFRP/GFRP rods	ILSS	[23]
	BFRP bars	Residual tensile strength	[24]
	GFRP rods	Bond strength	[25]
Eyring’s model	PA6,6, PC, CFRP	Creep failure time	[31]
Zhurkov’ model	PP	Fatigue strength	[32]
**Superposition principles**			
Time–temperature (TTSP)	Epoxy	Creep compliance	[28,33]
	Epoxy	Stress relaxation	[34]
	Filled epoxy	Stiffness/Relaxation modulus	[35]
	PMMA	Creep compliance	[36]
	Polyvinyl chloride, epoxy	Stress threshold of LVE	[37,38]
	Flax/vinylester	Creep compliance	[39]
	CFRP	Creep compliance	[40,41]
	CFRP, GFRP	Static/creep/fatigue strength	[42,43]
Time–moisture (TMSP)	Epoxy	Creep compliance	[28,33]
	Epoxy	Relaxation/storage modulus	[44,45,46,47,48]
	Epoxy-based compounds	Relaxation modulus	[49]
	Vinylester	Creep strain	[50]
	Polyester	Creep strain	[51]
	PA6, PA6,6	Storage modulus	[47,52]
	CFRP, GFRP	Fatigue strength	[53]
Time–stress (TSSP)	PA6	Creep strain	[54]
	PMMA	Creep compliance	[36,55,56]
	HDPE	Creep strain/lifetime	[57]
	Polycarbonate	Creep compliance	[58]
	PA6,6 fibres	Creep strain	[59]
	Glass/PA, PP, HDPE	Creep compliance	[60]
	HDPE/wood flour	Creep strain	[61]
	Graphite/epoxy FRP	Creep strain	[62]
	Kevlar yarns, PA6, epoxy	Creep strain (stepped isostress test)	[63,64,65]
Coupled			
TTSP + TMSP	Epoxy	Creep compliance	[28]
TTSP + TMSP	PA6,6	Storage modulus	[52]
TTSP + TMSP	Acrylate-based polymers	Storage modulus	[66]
TTSP + TMSP	CFRP, GFRP	Static/creep/fatigue strength	[53,67]
TTSP + TSSP	HDPE/wood flour	Creep strain	[61]
TASP+TMSP	Epoxy, polyester	Creep compliance, stress relaxation	[44,45]
TTSP+TASP	Epoxy	Relaxation modulus	[34,68]
TTSP+TASP+TSSP	PMMA	Creep strain	[55]
**Plasticity-controlled failure**	PP, PP/CNT, glass/PP, carbon/PEEK, PC/GF, PA6	Lifetime (tensile, creep, fatigue)	[69,70,71,72,73,74]
	PA6,6, PC, CFRP	Creep lifetime	[31]
**Parametric methods**	HDPE	Creep lifetime (Larson–Miller, Monkman–Grant)	[57]
	GFRP	Creep lifetime (Monkman–Grant)	[75]
	Rubber-bonded composite	Creep lifetime (Larson–Miller)	[76]
	Adhesive anchor in concrete	Creep lifetime (Monkman–Grant)	[77]
	Short fibre thermoplastics	Fatigue lifetime (Larson–Miller)	[78,79]

**Table 2:** A condensed list of recent works modelling fatigue under environmental impacts (*T* and *w* are associated with temperature and water effects, respectively).

Factor	Material/Testing Details	Prediction Method (s)	Author, Ref.
*T* *T + w*	Short fibre-reinforced thermoplastic composites, *R* = −1, 0.1, 3, 0.25–10 Hz	TTSP for *S–N* curves (dry and wet). *S–N* curve “normalisation”; strength degradation model with temperature-dependent parameters	Fatemi et al. [146,161,166]
*T* *T + w*	GFRP, four-point bending, *R* = 0.1, 4 Hz	TTSP for *S–N* curves (dry and wet)	Gagani et al. [26]
*T*	UD, braided, GFRP, CFRP; *R* = 0.1, 10, −0.8, −1; *f* = 3.3, 5, 10 Hz.	TTSP for *S*–*N* curves	Zhou et al. [133]
*T*	PP, PP/talc, PP/glass *R* = −1, 0.1, 0.3	Larson–Miller parametrisation for *S*–*N* curves; strength degradation model with temperature-dependent parameters.	Eftekhari et al. [78,79]
*T* *T + w*	CFRP, GFRP; tension, bending	*S–N* master curves by TTSP held for viscoelastic properties and static strength of the polymer matrix	Miyano et al. [42,53,67,90,162]
*T*	CFRP (AS4/PEEK) cross-ply, quasi-isotropic, *R* = 0.1; 5 Hz.	*S–N* curve “normalisation”	Jen et al. [164]
*T*	2.5D woven CFRP; *R* = 0.1; 10 Hz	*S–N* curve “normalisation”, residual stiffness model accounting temperature effect	Song et al. [165]
*T*	Weave GFRP *R* = 0.1; 5 Hz	Strength degradation model with two temperature-dependent parameters	Cormier et al. [136]
*w*	GFRP UD, biaxial, vinylester, R = 0.1, 5 Hz	Strength degradation model with parameters related to hydrothermal ageing time	Acosta et al. [169]
*w*	Plain-woven GFRP, R = 0.1, −0.52, 10; 5 Hz; seawater	Strength degradation model; *S–N* and CLT diagrams—model with ageing time-dependent parameters	Koshima et al. [167]
*T*	Cross-ply, quasi-isotropic, woven FRP composites	Cumulative fatigue damage model with temperature-dependent parameters determined in constant strain rate tests	Mivehchi et al. [149]
*T + w*	GFRP UD, *R* = 0.1, 2; 10 Hz; fresh, sea water	Stiffness degradation model with three material parameters dependent on environmental conditions; *S–N* curves	Tang et al. [151]
*T*	Weave woven CFRP/epoxy laminates; *R* = 0.1; 20 Hz	Stiffness degradation model with damage function dependent on temperature	Khan et al. [152]
*w*	CFRP woven; 3-point bending, *R* = 0.1, 1 Hz; seawater	Strain-life curves with two parameters depending on samples ageing	Prabhakar et al. [145]
*w*	Epoxy resin, *R* = 0.1	Viscoelastic/viscoplastic model with continuum damage accelerated by water plasticization	Rocha et al. [102]
*T,* UV	Triaxial CFRP laminates; *R* = −1; thermal cycles; 750 h UV	Stochastic analysis: Monte Carlo simulation for *S*–*N* curves of different guarantee areas depending on the ageing state	Mossalam et al. [137]
*w*	CFRP UD, cross-ply, bending, R = 0.1, 10 Hz, seawater	FEA modelling: virtual crack closure technique, water-induced accelerated crack propagation	Meng et al. [156]

## Data Availability

Not applicable.
